# The relationship between the intake of fruits, vegetables, and dairy products with hypertension: findings from the STEPS study

**DOI:** 10.1186/s40795-023-00756-3

**Published:** 2023-08-17

**Authors:** Mehran Nouri, Zainab Shateri, Mohebat Vali, Shiva Faghih

**Affiliations:** 1 Department of Community Nutrition, School of Nutrition and Food Sciences, Shiraz University of Medical Sciences, Shiraz, Iran; 2 Student Research Committee, Shiraz University of Medical Sciences, Shiraz, Iran; 3 Health Sciences Research Center, Mazandaran University of Medical Sciences, Sari, Iran; 4 Department of Nutrition and Biochemistry, School of Medicine, Ilam University of Medical Sciences, Ilam, Iran; 5 Nutrition Research Center, Shiraz University of Medical Sciences, Shiraz, Iran

**Keywords:** Fruit, Vegetable, Dairy, Hypertension, STEPS

## Abstract

**Background:**

The current research aimed to evaluate the relationship between fruit, vegetable (FV), and dairy consumption with the odds of developing hypertension based on nationwide Stepwise approach to surveillance (STEPS) data in Iran.

**Methods:**

This cross-sectional study was accomplished by the research center of non-communicable diseases (NCDs) in Tehran. In total, 29,378 individuals’ data were analyzed. Participants were classified into normal, elevated BP, stage I, and stage II hypertension according to systolic blood pressure (SBP) and diastolic blood pressure (DBP) examinations. Based on the STEPS questionnaire, the consumption of FVs and dairy products was evaluated. Multinomial logistic regression was applied to assess the relationship between the consumption of FVs and dairy products with hypertension.

**Results:**

The findings revealed that only fruit consumption (≥ 2 servings/day) was negatively related to stage I hypertension (odds ratio (OR) = 0.81; 95% confidence interval (CI): 0.69–0.95) in two servings per day and OR = 0.81; 95% CI: 0.68–0.96 in > two servings per day) in the adjusted model. There was no significant relationship between consuming vegetables and dairy products with elevated BP and hypertension.

**Conclusion:**

Our study showed that increasing fruit consumption was related to reducing hypertension odds. Regarding the consumption of dairy products and vegetables, no significant relationship was found with the odds of hypertension. More studies, especially cohorts, are needed to evaluate the impacts of FV and dairy products on the risk of hypertension.

## Introduction

Hypertension is an important preventable risk factor for cardiovascular diseases (CVDs), including heart failure, stroke, and myocardial infarction [1, 2]. Hypertension is considered as systolic blood pressure (SBP) equal to or more than 140 mmHg or diastolic blood pressure (DBP) equal to or more than 90 mmHg [3]. The hypertension prevalence is rapidly increasing [4]. The prevalence of this disease has been reported at 19.2% in Iranian samples [5].

Risk factors associated with hypertension include genetic factors, dietary factors, physical inactivity, and obesity [6]. Dietary patterns, as one of the lifestyle factors, can have an important effect on the continuous prevalence of hypertension [7].

Fruits and vegetables (FV) are an important part of the diet, containing vitamins, minerals, and folic acid that benefit endothelial function [8, 9]. Endothelial dysfunction is recognized as a hypertension risk factor [10]. Some studies have shown an inverse relationship between FV intake and hypertension [11, 12]. In contrast, other studies failed to show an effect of increased FV consumption on lowering the risk of hypertension [13, 14].

Overall, a healthy diet is vital for preventing hypertension [15]. However, whether dairy products should be included in such a diet is controversial [16]. Some epidemiological studies have shown an inverse relationship between dairy products and hypertension [17, 18]. The protective impacts of dairy products on hypertension can be caused by bioactive peptides such as lactotripeptides and micronutrients such as potassium, calcium, and magnesium [19, 20]. However, some studies have reported no association between dairy consumption and hypertension [21, 22].

Therefore, since there are inconsistent findings regarding the association between the consumption of FVs and dairy products with hypertension in different studies, and considering that no study with a large sample has been conducted on the Iranian population, the current study aimed to evaluate the relationship between the intake of FVs and dairy products with hypertension by applying data from the nationwide Stepwise approach to surveillance (STEPS) survey in Iran in 2016.

## Methods

### Sampling and design

This cross-sectional study was conducted by the Tehran University of Medical Sciences at the Non-Communicable Diseases (NCDs) Research Center [23]. This study used the STEPS questionnaire adapted from the World Health Organization (WHO) [24]. The validity and reliability of this questionnaire have already been evaluated [23]. This national and sub-national study was done in all provinces of Iran (except Qom) in 2016. The number of 30,541 participants was selected by cluster random sampling method based on each province’s population and fulfilled the STEPS questionnaire in the study’s first phase. The eligible participants selected based on aged ≥ 18 years and residing in Iran at the time of study. Also, participants were selected by postal code and national ID. In the next step, the history of NCD, medical risk factors, and data related to individuals’ lifestyles and socio-demographic characteristics were evaluated. In the next level, after a physical checkup, 30,042 participants were chosen. Ultimately, 29,378 participants were included in the final analysis (those whose SBP and DBP were assessed). In phases 2 and 3, anthropometric information and laboratory tests were evaluated. People over 18 years old were examined in the present study. Our study was confirmed by the Ethics Committee of Shiraz University of Medical Sciences (IR.SUMS.SCHEANUT.REC.1400.035).

### Measurements

SBP and DBP were measured in a sitting position for participants after five minutes of rest (BM 20, Beurer, Germany). This evaluation was repeated two times. The mean of evaluations was used for interpretation. Participants were classified into normal (SBP < 120 mmHg and DBP < 80 mmHg), elevated BP (SBP 120 to 129 mmHg or DBP 80 to 84 mmHg), stage I hypertension (SBP 130 to 139 mmHg or DBP 85 to 89 mmHg), and stage II hypertension (SBP ≥ 140 mmHg or DBP ≥ 90 mmHg) based on SBP and DBP examination [25]. Based on standard methods, height and weight were measured, and body mass index (BMI) was computed based on: weight (kg) / height (m^2^) [23].

The Global Physical Activity Questionnaire (GPAQ) was used to evaluate the participants’ physical activity levels. The data were converted to the metabolic equivalent of tasks (METs)-minutes per week for physical activity domains based on the GPAQ Instrument [23]. The details of physical activity calculation based on METs have been published in a previous study [26].

### Dietary factors

The following question was asked to find dietary intakes: “How many servings of dairy products or fruits or vegetables do you usually eat daily?”. One cup of dairy products, half a cup of cooked vegetables or one cup of raw vegetables, and one medium fruit was considered the amount of one serving. The consumption of dairy, vegetables, and fruits was categorized into:

Vegetable intake: less than 2, 2, 3, and more than 3 servings per day.

Fruit intake: less than 1, 1, 2, and > 2 servings per day.

Dairy product intake: <1, 1, 2, and > 2 servings per day [27].

### Statistical analysis

SPSS (version 25, SPSS Inc., Chicago IL, USA) was applied to analyze the data. A p-value < 0.05 was defined as statistically significant. Also, R software (version 3.0.2) was used for figures’ depictions. Baseline characteristics of this study were shown as median (interquartile range (IQR)) or percentage and means ± standard deviations (SDs). The chi-square test and analysis of variance (ANOVA) or Kruskal-Wallis test were used for categorical and continuous variables, respectively. Also, multinomial logistic regression was used to assess the relationship between dairy product, vegetable, and fruit consumption with blood pressure (BP) category. Participants were classified into normal (SBP < 120 mmHg and DBP < 80 mmHg), elevated BP (SBP 120 to 129 mmHg or DBP 80 to 84 mmHg), stage I hypertension (SBP 130 to 139 mmHg or DBP 85 to 89 mmHg), and stage II hypertension (SBP ≥ 140 mmHg or DBP ≥ 90 mmHg) based on SBP and DBP examination. In the adjusted model, gender, education, age, medication, occupation, marital status, area of residency, physical activity, wealth index (WI), and smoking history were controlled. The WI was calculated using the principal component analysis (PCA) method. Bartlett and Kaiser-Meier-Olkin (KMO) tests were utilized to reduce data. The WI was divided into five categories so that the first category represented the poorest group regarding income, and the last category presented the richest one [28].

## Results

**Fig. 1:**
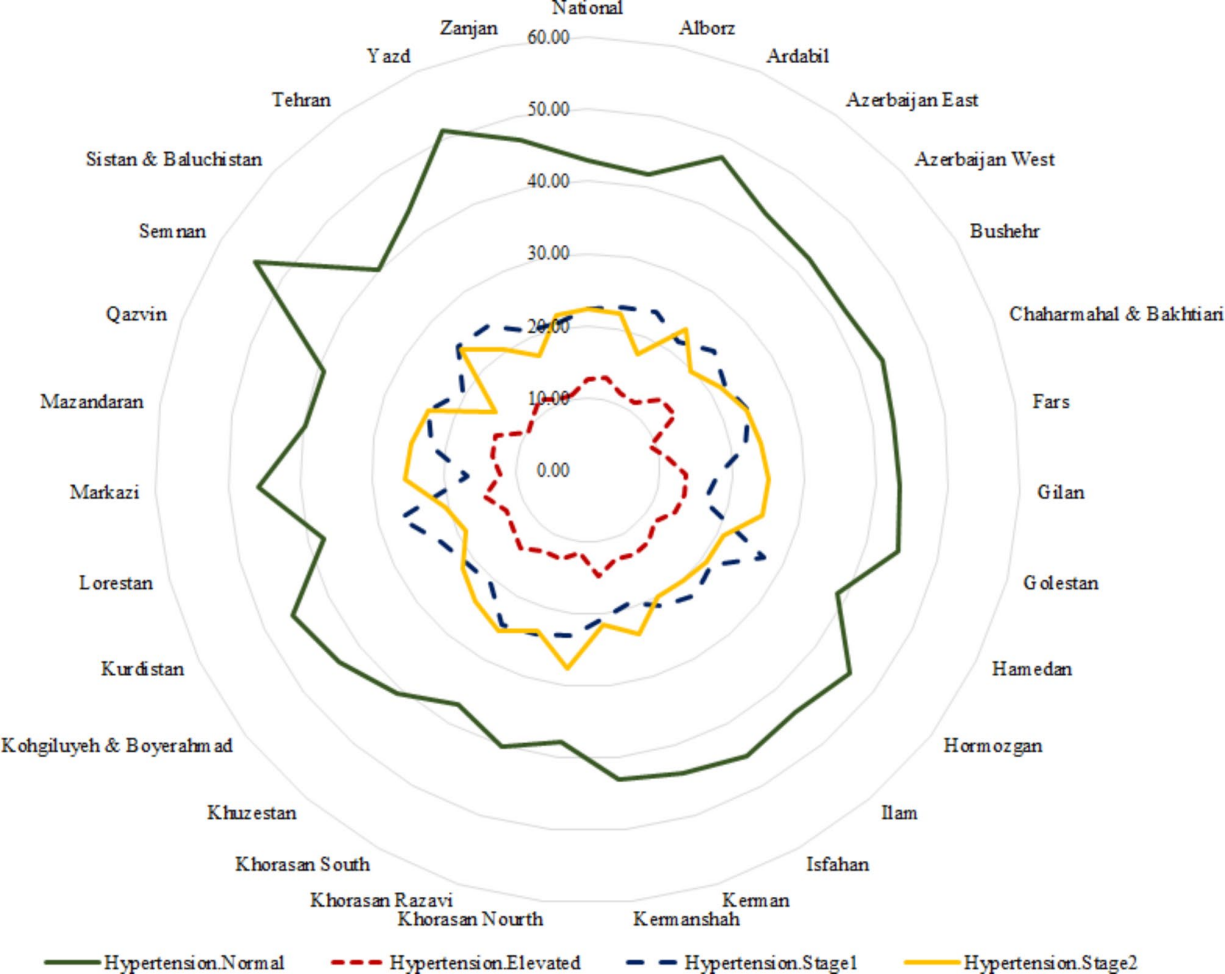
The prevalence of hypertension classification based on the national and subnational level

**Fig. 2:**
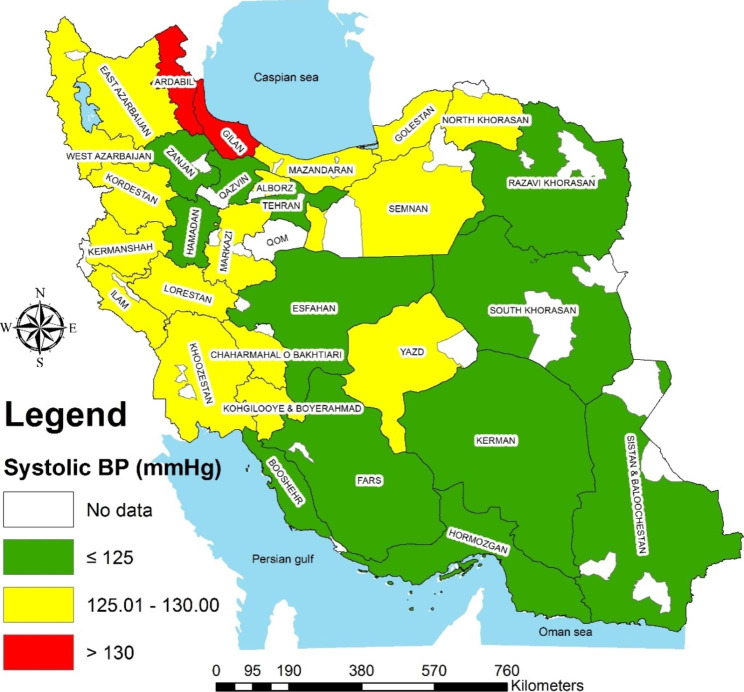
The distribution of systolic blood pressure at the level of the province

**Fig. 3:**
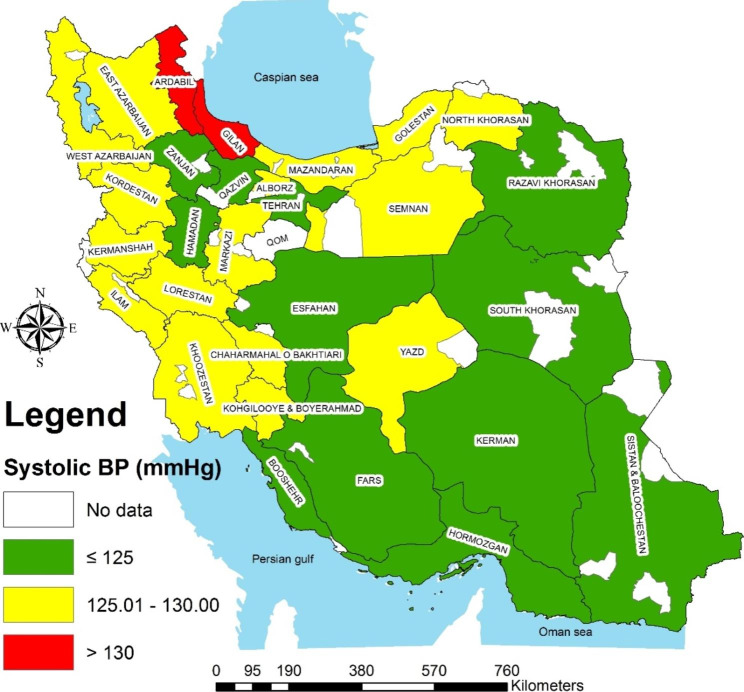
The distribution of diastolic blood pressure at the level of the province

In total, 29,378 individuals’ data were analyzed. Males comprised 52.3% of participants, and 29.6% lived in rural areas. The study participants’ mean age, education, and physical activity were 44.5 years, 8.2 years, and 1,684 MET/min/week, respectively (Table 1). Based on hypertension classification, there were significant differences in gender, area of sedentary, marital status, occupation, WI, age, BMI, physical activity, and education (P<0.001 for all). Also, the prevalence of normal hypertension, elevated, stage I, and II are presented in Fig. 1 (43.0%, 12.5%, 22.4%, and 22.1%, respectively) (distribution of hypertension at the subnational level is shown in Figs. 2 and 3).

**Table 1:** Socio-demographic characteristics of the study population

Variables	Normal (12,657)	Elevated (3,670)	Stage I (6,554)	Stage II (6,497)	Total (29,378)	P-value
Gender, female, %	54.0	50.5	52.3	50.2	52.3	**<0.001**
Area, rural, %	28.0	32.9	28.2	32.4	29.6	**<0.001**
Marital status, single, %	12.5	15.8	17.2	15.8	14.7	**<0.001**
Occupation						**<0.001**
Employee Worker Self-employed housewife Other	7.9 5.6 21.6 45.1 19.9	7.8 7.1 26.9 40.4 17.9	8.5 6.8 24.1 42.4 18.2	9.1 6.3 26.4 40.1 18.1	8.3 6.2 23.9 42.8 18.9	
Smoking, no, %	79.7	80.2	80.6	79.3	79.9	0.256
WI						**<0.001**
Poorest Poor Moderate Rich Richest	19.1 19.7 19.8 19.7 21.6	21.8 20.2 18.9 20.6 18.5	20.0 20.6 20.7 19.9 18.8	19.6 21.0 20.3 20.2 18.8	19.7 20.3 20.0 20.0 20.0	
Age, years	47.4 ± 17.1	42.5 ± 15.3	41.6 ± 15.2	42.9 ± 15.3	44.5 ± 16.2	**<0.001**
BMI (kg/m^2^)	26.9 ± 5.2	26.1 ± 4.9	26.2 ± 4.9	26.3 ± 5.1	26.5 ± 5.1	**<0.001**
Physical activity, MET/min/week	360.0 (1680.0)	456.0 (2100.0)	420.0 (1840.0)	480.0 (2160.0)	400.0 (1904.0)	**<0.001**
Education, years	8.0 (9.0)	9.0 (7.0)	10.0 (7.0)	9.0 (7.0)	9.0 (7.0)	**<0.001**

BMI, body mass index; MET, metabolic equivalent of task; WI, wealth index; SBP, systolic blood pressure; DBP, diastolic blood pressure; BP, blood pressure

Values are mean ± SD for continuous and percentage for categorical variables

BP category: normal (SBP < 120 mmHg and DBP < 80 mmHg), elevated BP (SBP 120 to 129 mmHg or DBP 80 to 84 mmHg), stage I hypertension (SBP 130 to 139 mmHg or DBP 85 to 89 mmHg), and stage II hypertension (SBP ≥ 140 mmHg or DBP ≥ 90 mmHg)

Also, the distribution of FV and dairy product consumption according to the classification of HTN is shown in Fig. 4A, 4B and 4C.

**Fig. 4A:**
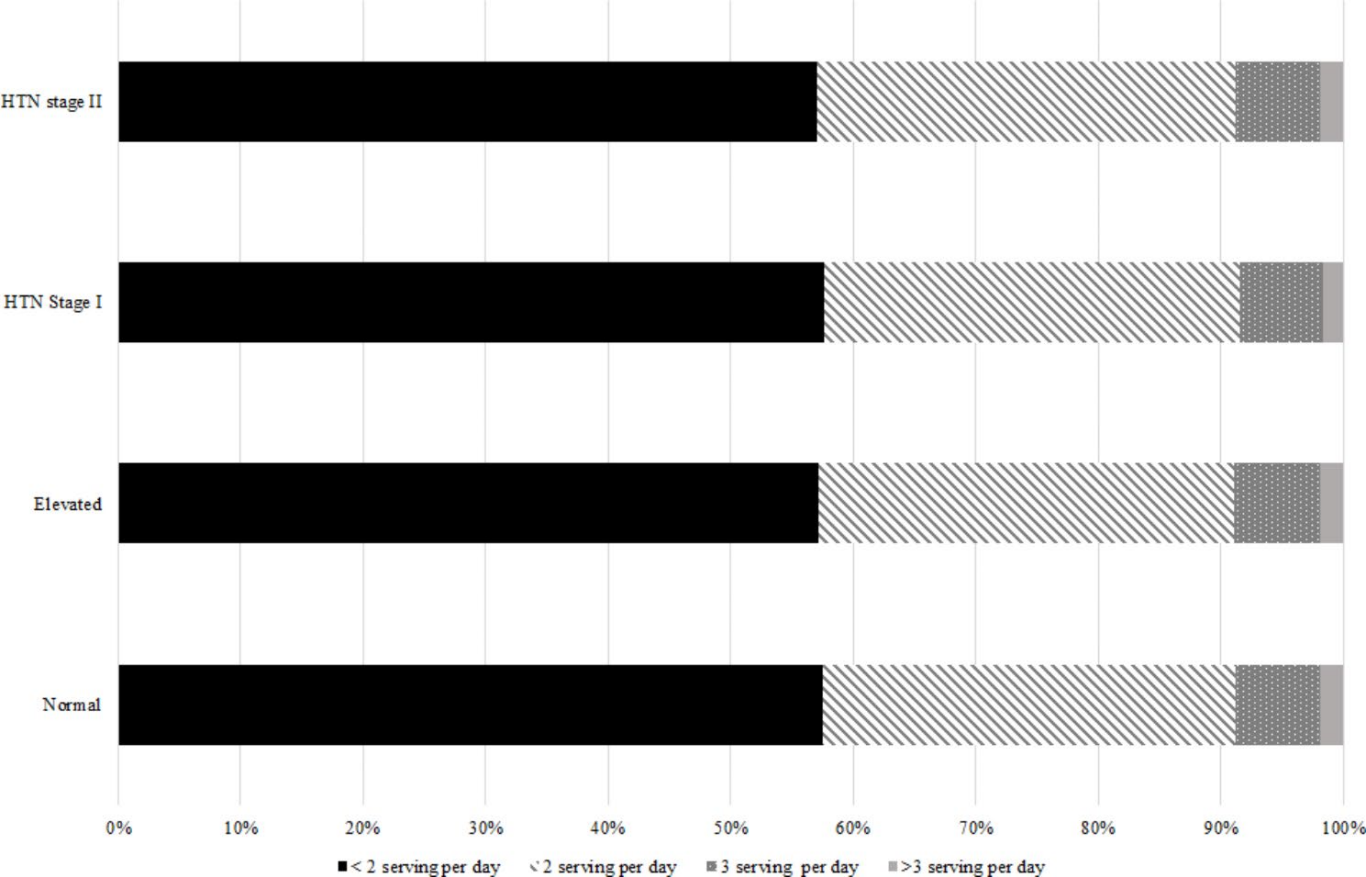
Distribution of vegetable consumption based on hypertension classification

**Fig. 4B:**
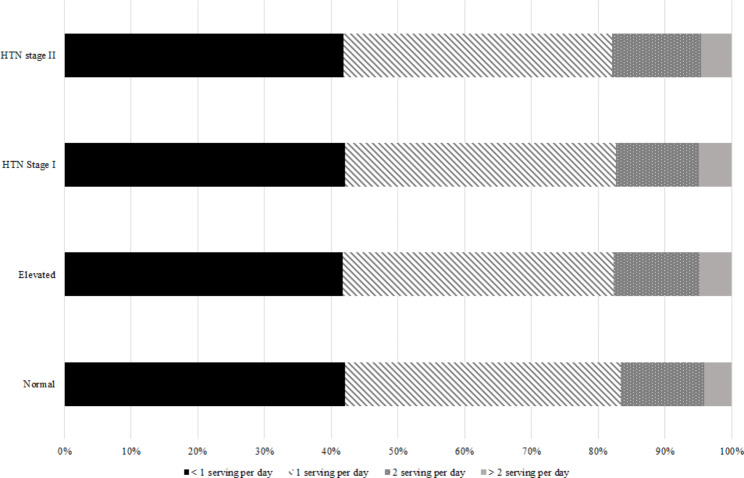
Distribution of fruit consumption based on hypertension classification

**Fig. 4C:**
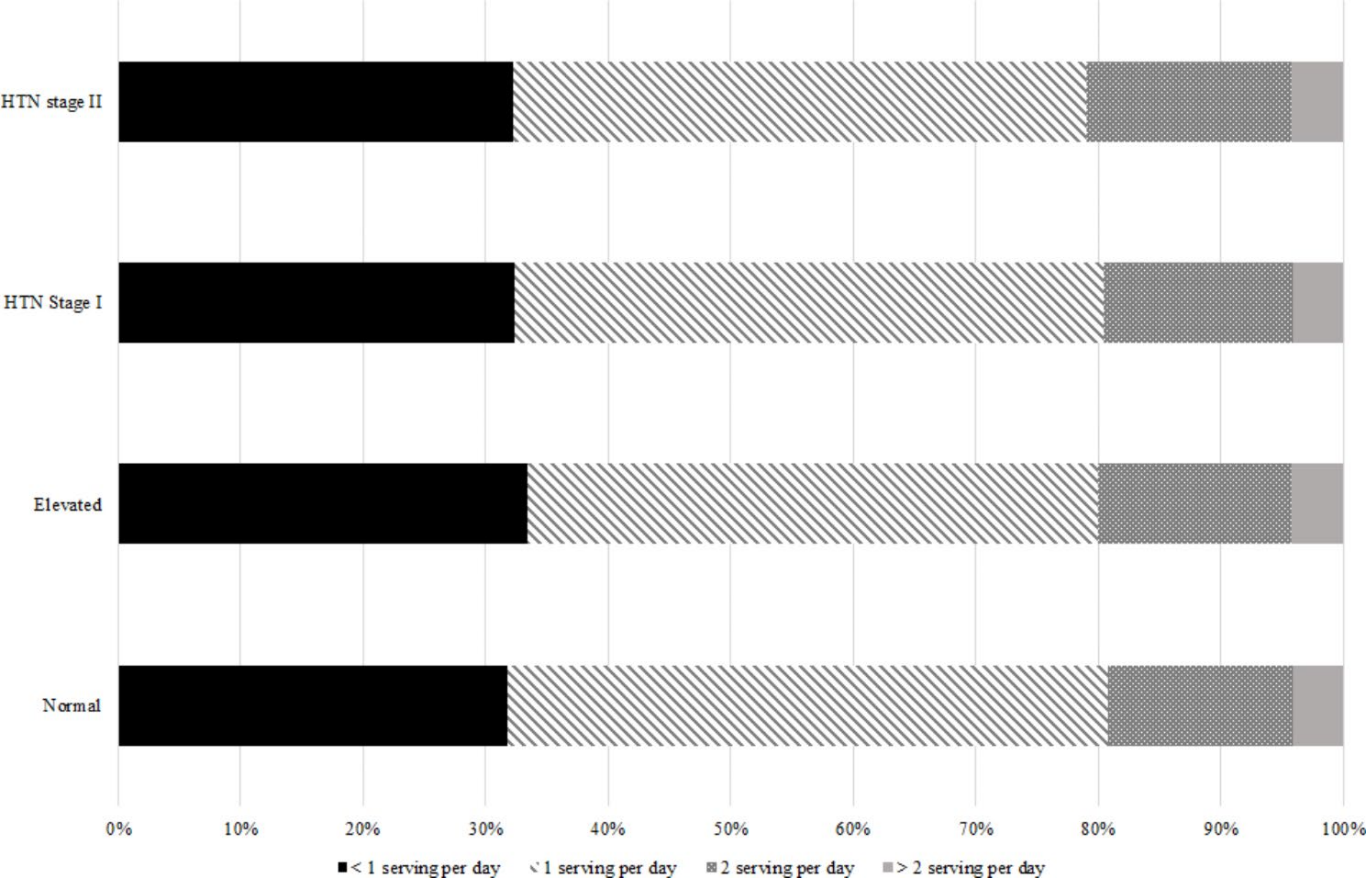
Distribution of dairy product consumption based on hypertension classification

According to Table 2 and Table 3, compared to less than one serving of fruit intake/day, we observed a reverse association between consumption of one, two, and more than two servings per day of fruits with the odds of stage I hypertension in the crude model (one serving – odds ratio (OR) = 0.82; 95% confidence interval (CI): 0.71–0.94–2 servings - OR = 0.80; 95% CI: 0.69–0.93 and more than two servings - OR = 0.80; 95% CI: 0.68–0.94). In the adjusted model, the odds of stage I hypertension was significantly decreased in participants who consumed two servings (OR = 0.81; 95% CI: 0.69–0.95) and more than two servings per day of fruits (OR = 0.81; 95% CI: 0.68–0.96) in comparison to less than one serving of fruit consumption. Also, neither crude nor adjusted models found any association between the consumption of vegetables and dairy products and the odds of hypertension.

**Table 2:** The association between vegetable, fruit, and dairy product consumption with BP classification in the crude model

Vegetable Intake				
Variables	< 2 servings	2 servings	3 servings	> 3 servings
Elevated vs. normal hypertension^*^	Ref.	0.98 (0.74–1.29)	1.00 (0.76–1.32)	1.13 (0.84–1.54)
Stage I hypertension vs. normal hypertension	Ref.	1.10 (0.87–1.38)	1.11 (0.88–1.39)	1.22 (0.95–1.57)
Stage II hypertension vs. normal hypertension	Ref.	0.97 (0.78–1.21)	1.00 (0.80–1.25)	1.11 (0.87–1.42)
**Fruit Intake**				
**Variables**	**< 1 serving**	**1 serving**	**2 servings**	**> 2 servings**
Elevated vs. normal hypertension	Ref.	0.85 (0.71–1.02)	0.85 (0.71–1.02)	0.88 (0.72–1.08)
Stage I hypertension vs. normal hypertension	Ref.	**0.82 (0.71–0.94)**	**0.80 (0.69–0.93)**	**0.80 (0.68–0.94)**
Stage II hypertension vs. normal hypertension	Ref.	0.88 (0.76–1.02)	0.86 (0.74-1.00)	0.94 (0.80–1.11)
**Dairy Product Intake**				
**Variables**	**< 1 serving**	**1 serving**	**2 servings**	**> 2 servings**
Elevated vs. normal hypertension	Ref.	1.02 (0.84–1.24)	0.92 (0.76–1.11)	1.02 (0.83–1.25)
Stage I hypertension vs. normal hypertension	Ref.	1.00 (0.86–1.17)	0.96 (0.83–1.12)	1.00 (0.85–1.18)
Stage II hypertension vs. normal hypertension	Ref.	0.96 (0.82–1.12)	0.91 (0.78–1.06)	1.05 (0.89–1.23)

SBP, systolic blood pressure; DBP, diastolic blood pressure; BP, blood pressure

BP category: normal (SBP < 120 mmHg and DBP < 80 mmHg), elevated BP (SBP 120 to 129 mmHg or DBP 80 to 84 mm Hg), stage I hypertension (SBP 130 to 139 mm Hg or DBP 85 to 89 mmHg), and stage II hypertension (SBP ≥ 140 mmHg or DBP ≥ 90 mmHg)

These values are odds ratio (95% CIs) and obtained from multinomial regression

* Reference category

**Table 3:** The association between vegetable, fruit, and dairy product consumption with BP classification in the adjusted model

Vegetable Intake				
Variables	< 2 servings	2 servings	3 servings	> 3 servings
Elevated vs. normal hypertension^*^	Ref.	0.92 (0.68–1.26)	0.92 (0.68–1.26)	1.09 (0.78–1.53)
Stage I hypertension vs. normal hypertension	Ref.	0.99 (0.77–1.28)	0.99 (0.76–1.28)	1.11 (0.84–1.47)
Stage II hypertension vs. normal hypertension	Ref.	0.90 (0.70–1.16)	0.91 (0.71–1.17)	1.04 (0.79–1.38)
**Fruit Intake**				
**Variables**	**< 1 serving**	**1 serving**	**2 servings**	**> 2 servings**
Elevated vs. normal hypertension	Ref.	0.90 (0.74–1.10)	0.88 (0.72–1.07)	0.89 (0.71–1.10)
Stage I hypertension vs. normal hypertension	Ref.	0.85 (0.73-1.00)	**0.81 (0.69–0.95)**	**0.81 (0.68–0.96)**
Stage II hypertension vs. normal hypertension	Ref.	0.93 (0.79–1.10)	0.90 (0.76–1.06)	0.97 (0.81–1.16)
**Dairy Product Intake**				
**Variables**	**< 1 serving**	**1 serving**	**2 servings**	**> 2 servings**
Elevated vs. normal hypertension	Ref.	1.09 (0.88–1.36)	1.01 (0.81–1.25)	1.02 (0.81–1.29)
Stage I hypertension vs. normal hypertension	Ref.	1.03 (0.86–1.23)	0.97 (0.81–1.16)	1.00 (0.83–1.21)
Stage II hypertension vs. normal hypertension	Ref.	1.03 (0.86–1.23)	0.95 (0.80–1.14)	1.05 (0.87–1.27)

SBP, systolic blood pressure; DBP, diastolic blood pressure; BP, blood pressure

BP category: normal (SBP < 120 mmHg and DBP < 80 mmHg), elevated BP (SBP 120 to 129 mmHg or DBP 80 to 84 mmHg), stage I hypertension (SBP 130 to 139 mmHg or DBP 85 to 89 mmHg), and stage II hypertension (SBP ≥ 140 mmHg or DBP ≥ 90 mmHg)

Adjusted for gender, age, BMI, marital status, physical activity, education, occupation, area of residency, wealth index, and smoking history

These values are odds ratio (95% CIs) and obtained from multinomial regression

* Reference category

## Discussion

In the crude model, we found that the odds of developing stage I hypertension was lower in people who consumed more fruits (more than one serving). Also, in the adjusted model, only fruit consumption (≥ 2 servings/day) was negatively associated with stage I hypertension. Moreover, there was no significant relationship between consuming vegetables and dairy products with elevated BP and hypertension.

The results of a cross-sectional study indicated that a plant-based diet had a beneficial effect on hypertension [7]. There are several mechanisms for the association between FV intake and hypertension risk. FV are high in vitamin C, magnesium, folic acid, potassium, carotenoid, and flavonoid, which can help reduce BP [6]. Also, FV intake cause an increasing intake of fiber and decreasing fat intake [29]. It has been shown that people who consumed more dietary fiber were less prone to hypertension [30]. Moreover, increased consumption of FV may decrease BP by improving anthropometric parameters of the body, such as reducing overweight or obesity [31].

The findings showed the inverse relationship between fruit consumption and hypertension. The current study’s findings were consistent with some other studies. A study by Kim et al. revealed that a higher fruit intake was inversely correlated to hypertension risk in older and middle-aged adults [32]. Also, Borgi et al. reported that higher whole fruit intake decreased the hypertension risk [33]. Moreover, a systematic review and meta-analysis study showed that increasing fruit consumption by almost 300 gr/day reduced hypertension risk by 7% [34].

As previously mentioned, there was no correlation between the consumption of vegetables and hypertension odds in the current study. A study by Kim et al. proved no significant association between vegetable intake and hypertension [32]. Also, three prospective cohort studies showed no significant association between vegetable consumption and hypertension risk [33]. Contrary to the present study’s findings, Wang et al. indicated a negative relationship between vegetable intake and hypertension [35]. Further, Utsugi et al. reported a negative relationship between vegetable consumption and hypertension [11]. The difference in the population, samples, and definitions of hypertension in the mentioned studies and the present study can be the reason for their inconsistent results with the current research.

A study by Muslimi et al. on Iranian samples showed that 53.33% of the participants fried vegetables moderately [36]. Also, Djazayery et al. showed that 77% of Iranian women fried or boiled vegetables for a long time [37]. On the other hand, Miglio et al. indicated that frying vegetables reduced their antioxidant compounds. Also, their results showed that frying and boiling vegetables decreased their nutritional quality [38]. Cooking practices of vegetables in Iran may be the reason for the present study’s lack of a relationship between vegetable consumption and hypertension.

The present study found no significant relationship between dairy product consumption and elevated BP and hypertension. In line with our results, research by Villaverde et al. demonstrated no significant association between the overall intake of dairy products and hypertension in a prospective cohort study [39]. Also, Heraclides et al. reported that specific dairy subgroups (fermented, low-fat, and full-fat) or total dairy products were unrelated to BP and hypertension after ten years of follow-up [40]. While in a systematic review and meta-analysis study on cohort studies, an inverse and significant relationship was found between the consumption of low-fat dairy products and hypertension risk. Also, a study found a nonlinear relationship between total dairy and hypertension [41]. The relationship between different types of dairy products and hypertension risk has been examined. In a 15-year prospective study by Steffen et al., cheese consumption did not significantly correlate with hypertension [42]. However, a study found an inverse relationship between yogurt consumption and hypertension risk [43]. Besides recommendations on other food groups’ intake, the Dietary Approaches to Stop Hypertension (DASH) diet has emphasized intaking more than two servings/day of low-fat dairy products [44]. Most clinical trial studies have shown the role of the DASH diet in lowering BP [45, 46]. The present study assessed total dairy product consumption without separating low-fat versus high-fat dairy products. It may be the reason for the non-association between dairy products and hypertension. Dairy products are the primary sources of calcium, which has a beneficial role in reducing BP in patients with hypertension [47, 48]. A systematic review study by Cormick et al. revealed that by increasing calcium intake, SBP and DBP decreased by 1.37 mm Hg and 1.45 mmHg, respectively. This effect was greater with doses of calcium > 1000 mg daily [49]. However, the impact of dairy products on hypertension is not apparent [50, 51], especially in the elderly, as the BP-lowering response to dairy consumption may vary with age [52].

Among the strengths of the current research, we can mention the large sample size and the adjustment of the effect of confounders. Since this study was a cross-sectional study, it is impossible to discuss the effect mechanisms of fruit and dairy product intake on hypertension. Also, due to the unavailability of data on low-fat and high-fat dairy products, it was impossible to investigate their effect separately on hypertension. In addition, the unavailability of data on energy and salt intake was another limitation of the present study.

## Conclusion

In conclusion, our study showed that increasing fruit consumption was related to reducing hypertension odds. Regarding the consumption of dairy products and vegetables, no significant relationship was found with the odds of hypertension. More studies, especially cohorts, are needed to assess the impacts of FV and dairy products on the risk of hypertension.

## Data Availability

To protect study participant privacy, data cannot be shared openly, but data are available through a reasonable request from the corresponding author.
